# Monocular Depth Estimation with Joint Attention Feature Distillation and Wavelet-Based Loss Function

**DOI:** 10.3390/s21010054

**Published:** 2020-12-24

**Authors:** Peng Liu, Zonghua Zhang, Zhaozong Meng, Nan Gao

**Affiliations:** 1 State Key Laboratory of Reliability and Intelligence of Electrical Equipment, Hebei University of Technology, Tianjin 300130, China; niatdlut@163.com (P.L.); zhaozong.meng@hebut.edu.cn (Z.M.); ngao@hebut.edu.cn (N.G.); 2 School of Mechanical Engineering, Hebei University of Technology, Tianjin 300130, China; 3 Key Laboratory of Intelligent Data Information Processing and Control of Hebei Province, Tangshan University, Tangshan 063000, China

**Keywords:** monocular depth estimation, feature distillation, joint attention, loss function

## Abstract

Depth estimation is a crucial component in many 3D vision applications. Monocular depth estimation is gaining increasing interest due to flexible use and extremely low system requirements, but inherently ill-posed and ambiguous characteristics still cause unsatisfactory estimation results. This paper proposes a new deep convolutional neural network for monocular depth estimation. The network applies joint attention feature distillation and wavelet-based loss function to recover the depth information of a scene. Two improvements were achieved, compared with previous methods. First, we combined feature distillation and joint attention mechanisms to boost feature modulation discrimination. The network extracts hierarchical features using a progressive feature distillation and refinement strategy and aggregates features using a joint attention operation. Second, we adopted a wavelet-based loss function for network training, which improves loss function effectiveness by obtaining more structural details. The experimental results on challenging indoor and outdoor benchmark datasets verified the proposed method’s superiority compared with current state-of-the-art methods.

## 1. Introduction

Depth estimation is a fundamental computer vision task and is in high demand for manifold 3D vision applications, such as scene understanding [1], robot navigation [2,3], action recognition [4], 3D object detection [5], etc. Monocular depth estimation (MDE) is a more affordable solution for depth acquisition due to extremely low sensor requirements, compared with common depth sensors, e.g., Microsoft’s Kinect or stereo images. However, MDE is ill-posed and inherently ambiguous due to one-too-many mapping from 2D to 3D and remains a very challenging topic.

Classical approaches often design hand-crafted features to deduce depth information, but hand-crafted features have no generality across different real-world scenes. Hence, classical approaches have considerable difficulty in acquiring reasonable accuracy. Deep convolutional neural network (DCNN) architectures could be considered as the effective reconstruction methods for many applications with ill-posed problem properties [6,7,8]. Powerful feature generalization and representation has become available recently through DCNN, which have been successfully introduced to MDE and demonstrated superior performances to the classical approaches [9].

**Figure 1:**
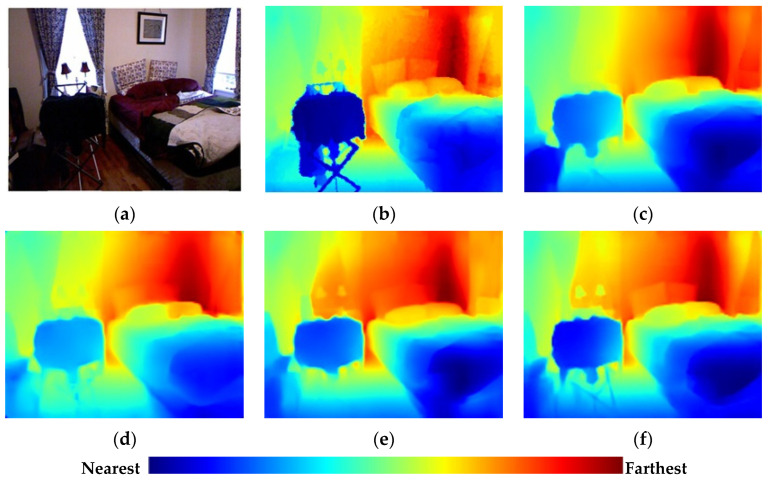
A depth estimation example: (**a**) RGB image; (**b**) ground truth depth map; and (**c**–**f**) depth maps by Chen et al. [20], Alhashim et al. [17], Hu et al. [22], and the proposed method. We set colors of all indoor depth maps in our work according to the distance as the color bar above.

Most DCNN-based MDE methods are based on encoder–decoder architecture. Standard DCNN originally designed for the image classification task are selected as encoders, e.g., ResNet [10], DenseNet [11], SENet [12], etc. These encoders gradually decrease the feature map spatial resolution by pooling while learning the rich feature representation. Since feature map resolution increases during decoding, various deep-learning methods have been adopted to provide high-quality estimations, including skip connection [13,14,15,16,17], multiscale feature extraction [18,19,20,21,22], attention mechanism [23,24,25,26], etc. Although great improvements have been achieved for MDE methods, reconstructing the depth for fine-grain details still requires further improvements, as shown in Figure 1.

The current methods struggle to precisely recover large-scale geometry regions (walls) and local detail regions with rich structural information (boundaries and small parts) simultaneously, because the methods still lack the sufficient flexibility and discriminative modulation ability to handle regions with different feature information during up-sampling. This insufficiency limits the feature representation and significantly reduces the estimation accuracy in many cases.

Another area for improvement is the loss function design. Several loss function terms are commonly combined to construct loss functions for predicting a better-quality depth. Various weight-setting methods for the loss function terms have been proposed to balance the training process [27,28,29], but how to enhance loss function effectiveness for fixed loss term combinations remains an open question.

Therefore, we proposed a new DCNN to settle this issue. We designed an attention-based feature distillation block (AFDB) to address the insufficiency above and integrate it into each up-sampling process in the decoder. To our best knowledge, this is the first time feature distillation has been introduced to MDE. The AFDB enriches feature representation through a series of distillation and residual asymmetric convolution (RAC) layers. We also propose a joint attention module (JAM) to adaptively and simultaneously rescale features depending on the channel and spatial contexts. The designed AFDB incorporates the proposed JAM, providing flexible and discriminative modulation to handle the features.

We also designed a wavelet-based loss function to enhance the loss function effectiveness by combining the multiple loss function with discrete wavelet transform (DWT). The estimated depth map is first divided into many patches using DWT at various frequencies, highlighting high-frequency information from depth map edge areas. The loss for each patch is then reasonably combined to generate the final loss. The experimental results verified that this loss function modification could significantly improve various metrics on benchmark datasets.

Our main contributions are summarized as follows:

- A novel AFDB was designed for the proposed DCNN-based MDE method by combining feature distillation and joint attention mechanisms to boost discriminative modulation for feature processing.
- A wavelet-based loss function was adopted to optimize the training by highlighting the structural detail losses and, hence, improve the estimation accuracy.
- The proposed network was superior to most state-of-the-art MDE methods on two public benchmark datasets: NYU-Depth-V2 and KITTI.

## 2. Related Works

We discuss and summarize supervised DCNN-based MDE methods in Section 2.1 and briefly review the related techniques, i.e., attention mechanism, feature distillation, and loss function design, in Section 2.2, Section 2.3 and Section 2.4, respectively.

### 2.1. Supervised DCNN-Based MDE Methods

The Supervised DCNN-based MDE methods utilize the DCNN to realize the nonlinear mapping from the RGB image to the depth map. The Supervised DCNN-based methods have become significantly efficient for MDE, with many publicly available RGB and depth map (RGBD) datasets, due to their powerful feature generalization and representation. Eigen et al. [30] proposed a multiscale deep network for MDE that included coarse and fine-scaled network pathways with skip connections between the corresponding layers. Laina et al. [31] used ResNet architecture and several up-projection operators to attain the final depth maps. Cao et al. [32] designed a fully convolutional deep residual network that explicitly considered the long tail distribution of the ground truth depth and regarded the MDE problem as a pixel-wise classification task.

Repeated pooling while learning the rich-feature representations for supervised DCNN-based models inevitably reduces the feature map spatial resolution, which poorly influences the fine-grain depth estimation. Li et al. [33] and Zheng et al. [34] integrated hierarchical depth features to settle this problem. They combined different resolution depth features with up-convolution to realize a coarse-to-fine process. Godard et al. [14] and Liu et al. [13] used skip connection to aggregate feature maps in lower layers, with same resolution feature maps in deeper layers. Other studies [18,19,20,21,22] have aggregated multiscale contexts to improve prediction performances. For example, Fu et al. [18] applied dilated convolution with multiple dilation rates to extract multiscale features and, subsequently, developed a full-image encoder to capture image level features, Zhao et al. [19] employed image super-resolution techniques to generate multiscale features, and Chen et al. [20] proposed an adaptive dense feature aggregation module to aggregate effective multiscale features to infer scene structures.

Several recent multitask learning methods [35,36,37,38,39,40] have been successfully introduced for MDE by estimating depth maps with other information, such as semantic segmentation labels, surface normals, super pixels, etc. For example, Eigen and Fergus [35] combined semantic segmentation, surface normal, and depth estimation cues to build a single DCNN. This single architecture simplifies implementing a system that requires multiple prediction tasks. Ito et al. [36] proposed a 3D representation for semantic segmentation and depth estimation from a single image. Lin et al. [37] proposed a hybrid DCNN to integrate semantic segmentation and depth estimation into a unified framework. Although multitask learning methods can boost estimation performances, the required multibranch design in the decoder increases the model parameters and reduces the running speed.

### 2.2. Attention Mechanism

The attention mechanism can enhance the network representation by increasing the model sensitivity to informative and important features. This has been widely adopted for MDE. For example, Chen et al. [23] enhanced the feature discrimination by designing an attention-based context fusion network to extract image and pixel-level context information, Li et al. [24] applied a channel-wise attention mechanism to extract discriminative features for each resolution, Wang et al. [25] used joint attention mechanisms in their framework to improve the presentation for highest level of feature maps, Chen et al. [15] proposed spatial attention and global context blocks to extract features by blending cross-channel information, and Huynh et al. [41] proposed a guiding depth estimation to favor planar structures by incorporating a nonlocal coplanarity constraint with a nonlocal attention mechanism.

### 2.3. Feature Distillation

Feature distillation is a recently developed method that has been efficiently applied to super-resolution tasks. The method usually adopts channel splitting to distill feature maps and gain more efficient information. Hui et al. [42] first proposed a feature distillation network to aggregate long and short path features. Hui et al. [43] further advanced the concept and constructed a lightweight cascaded feature multi-distillation block by combining distillation with selective fusion operation. The selective fusion was implemented by their proposed contrast-aware attention layer. Liu et al. [44] recently proposed a lightweight residual feature distillation network using a shallow residual block and multiple feature distillation connections to learn more discriminative representations. The proposed model was the winning solution for the advances in image manipulation 2020 (AIM2020) constrained image super-resolution challenge [45].

### 2.4. Loss Function Design

Learning in DCNNs is essentially an optimization process, i.e., a neural network adjusts the network weights depending on the loss function value. Therefore, the loss function is important for generating the final estimation model. Many previous studies combined multiple loss terms to build the loss function. However, some loss terms can be ignored during training when many are included, and an adaptive weight adjustment strategy is also required to balance the contribution from each loss term, since they reduce at different rates. Jiang et al. [27] proposed an adaptive weight allocation method based on a Gaussian model for their proposed hybrid loss function. Liu et al. [28] proposed an effective adaptive weight adjustment strategy to adjust each loss term’s weight during training. Lee et al. [29] proposed a loss rebalancing algorithm to initialize and rebalance weights for loss terms adaptively during training. Yang et al. [46] adopted DWT to reform the structural similarity (SSIM) loss [47] and achieved improved reconstructions. These methods were proposed to enhance the loss function effectiveness under fixed loss term combinations.

Although great improvements have been achieved for MDE methods, reconstructing the depth for fine-grain details still requires further improvements. Our proposed method employed a single-task encoder–decoder architecture that has fewer model parameters and faster running speed compared with the multitask learning architecture. We efficiently integrated feature distillation and joint attention mechanisms in the decoder to further boost the discriminative modulation for feature processing. We also combined multiple loss functions with DWT to enhance the loss function effectiveness.

## 3. Proposed Method

This section describes the proposed MDE method. Section 3.1 and Section 3.2 discuss the network architecture and provide details for the proposed AFDB, respectively. Section 3.3 details the proposed wavelet-based loss function.

### 3.1. Network Architecture

**Figure 2:**
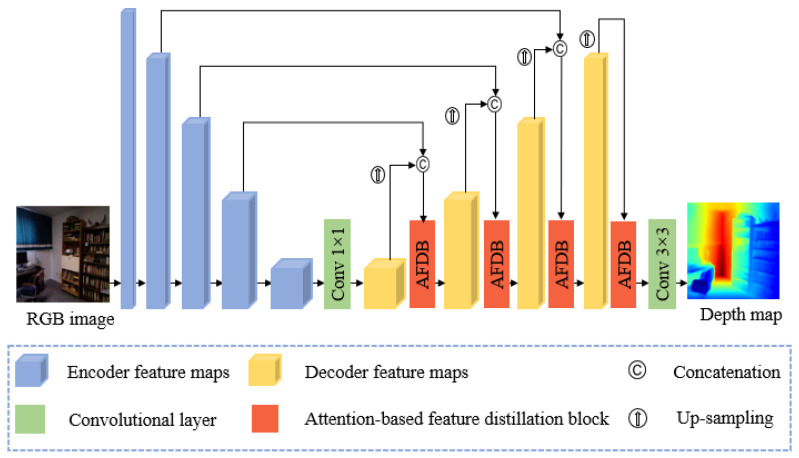
Proposed network architecture.

Figure 2 shows the proposed network architecture. We use a standard encoder–decoder architecture with skip connections between same resolution layers. The encoder is modified from the standard DCNN that was originally designed for image classification by removing the final average pooling and fully connected layers. In the decoding stage, we first attached a 1 × 1 convolutional layer to the top of the encoder for feature reduction. We concatenated up-sampled feature maps in the decoder with feature maps from the encoder that have the same resolution to enrich the feature representation and provide flexible and discriminative modulation for the feature maps. The concatenated feature maps were refined using the proposed AFDB. After gradually recovering the feature maps back to the expected depth map resolution, the AFDB output was fed into a 3 × 3 convolutional layer to derive the final estimation.

### 3.2. Attention-Based Feature Distillation

**Figure 3:**
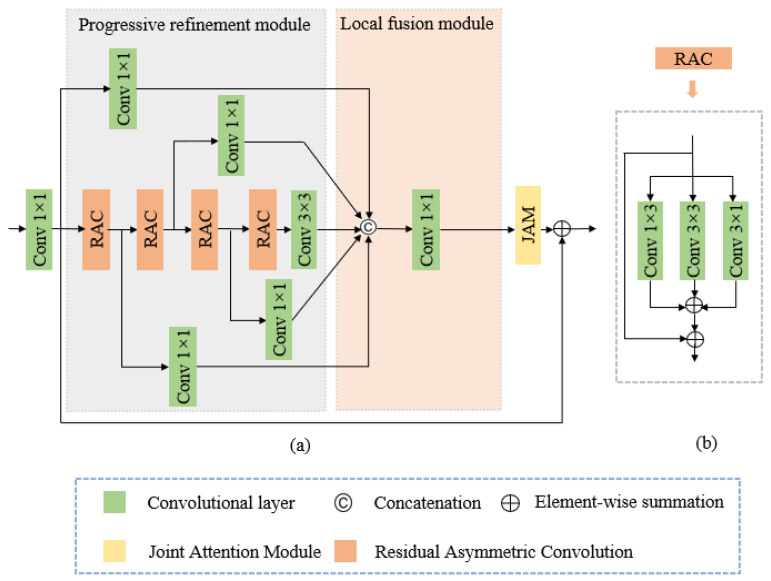
Proposed AFDB design with a four-step distillation example: (**a**) AFBD and (**b**) RAC structures.

Figure 3 shows the proposed AFDB to enrich the feature representation and improve the flexible and discriminative modulation during up-sampling in the decoder. The first 1 × 1 convolutional layer reduces the concatenated feature map channels from the encoder and decoder with the same resolution. The subsequent block with a residual connection includes the progressive refinement, local fusion, and joint attention modules.

The progressive refinement module enriches the feature representation through several distillation and feature refinement steps. The local fusion module is a commonly employed structure that includes concatenation and a 1 × 1 convolutional layer, providing local feature reduction and fusion for all branch outputs from the progressive refinement module. The JAM further enhances the feature discriminative modulation by fully considering the feature channel and spatial contexts.

The proposed AFDB was modified from the feature distillation block structure proposed by [44], incorporating two improvements. We replaced the shallow residual block of [44] with the RAC in the progressive refinement module, which efficiently enhanced the model robustness to rotational distortions in image classification [48]. We effectively integrated a channel attention branch in parallel to the original contrast aware attention layer, enhancing the discriminative modulation for the block.

#### 3.2.1. Progressive Refinement Module

Figure 3a shows the proposed progressive refinement module structure. Each step uses a 1 × 1 convolutional layer to distill some features and an RAC layer to further refine the remaining features simultaneously. The RAC comprises an asymmetric convolution with skip connections, where the asymmetric convolution comprises three parallel layers with 3 × 3, 3 × 1, and 1 × 3 kernels. The outputs are summed to enrich the feature representation.

Given the input features $F_{\mathrm{in}}$ for the progressive refinement block and four-step distillation, the procedure can be described as

(1) $$F_{{\mathrm{ref}}_{1}},F_{{\mathrm{dis}}_{1}}={\mathrm{Split}}_{1}\left(F_{\mathrm{in}}\right),$$

(2) $$F_{{\mathrm{ref}}_{2}},F_{{\mathrm{dis}}_{2}}={\mathrm{Split}}_{2}\left(F_{{\mathrm{ref}}_{1}}\right),$$

(3) $$F_{{\mathrm{ref}}_{3}},F_{{\mathrm{dis}}_{3}}={\mathrm{Split}}_{3}\left(F_{{\mathrm{ref}}_{2}}\right),$$

and

(4) $$F_{{\mathrm{ref}}_{4}},F_{{\mathrm{dis}}_{4}}={\mathrm{Split}}_{4}\left(F_{{\mathrm{ref}}_{3}}\right),$$

where ${\mathrm{Split}}_{i}$ denotes the i-th channel splitting operation, which includes a 1 × 1 convolutional layer to generate the distilled features $F_{{\mathrm{dis}}_{i}}$ and a 3 × 3 convolutional layer to generate the refined features $F_{{\mathrm{ref}}_{i}}$, which will be further processed by succeeding layers. Distilled feature channels are half the dimensionality of the original.

After the four-step operation, we use a 3 × 3 convolutional layer to further filter the last RCAB:

(5) $$F_{\mathrm{fil}}=W_{\mathrm{fil}}^{3\;\times \;3}\left(F_{{\mathrm{ref}}_{4}}\right),\;$$

where W denotes convolution.

The local fusion procedure can be expressed as

(6) $$F_{\mathrm{LF}}=W_{LF}^{1\;\times \;1}\left(\mathrm{Concat}(F_{\mathrm{fil}},F_{{\mathrm{dis}}_{1}},F_{{\mathrm{dis}}_{2}},F_{{\mathrm{dis}}_{3}},F_{{\mathrm{dis}}_{4}}\right)),$$

where Concat denotes concatenation.

#### 3.2.2. Joint Attention Module

**Figure 4:**
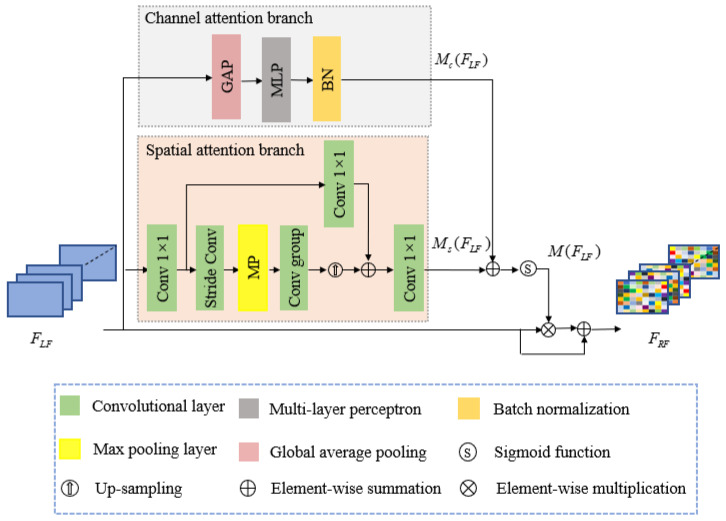
Proposed joint attention module (JAM) structure.

Figure 4 shows the proposed JAM structure, inspired by lightweight joint attention modules [49] that infer attention maps along the channel and spatial dimensions simultaneously, to further enhance the feature discriminative modulation. We adopted a residual connection and joint attention mechanism to facilitate the gradient flow. The JAM produces a 3D attention map for the input feature maps by combining parallel channel and spatial attention branches. Thus, JAM can refine feature maps and enhance the feature representation while fully considering the channel and spatial contexts.

Figure 4 shows that, for a given input feature map $F_{\mathrm{LF}}$, i.e., the local fusion module output, we simultaneously compute the channel attention $M_{c}\left(F_{\mathrm{LF}}\right)$ and spatial attention $M_{s}\left(F_{\mathrm{LF}}\right)$ in the channel and spatial attention branches, respectively. The joint 3D attention map $M\left(F_{\mathrm{LF}}\right)$ is then computed as

(7) $$M\left(F_{\mathrm{LF}}\right)=\sigma \left(M_{c}\left(F_{\mathrm{LF}}\right)+M_{s}\left(F_{\mathrm{LF}}\right)\right),$$

where σ denotes the sigmoid function. The refined feature maps are

(8) $$F_{\mathrm{RF}}=F_{\mathrm{LF}}+F_{\mathrm{LF}}⊗M\left(F_{\mathrm{LF}}\right),$$

where ⊗ denotes element-wise multiplication.

The channel attention $M_{c}\left(F_{\mathrm{LF}}\right)$ exploits the inter-channel relationships for the feature maps, which mainly includes three steps (Figure 4):

1. Global average pooling on the input feature maps to fetch global information for each channel.
2. Multilayer perceptron with one hidden layer to predict the attention across the computed channels.
3. Batch normalization layer to adjust the scale with another spatial branch output.

The procedure can be described mathematically as

(9) $$M_{c}\left(F_{\mathrm{LF}}\right)=BN\left(MLP\left(GAP\left(F_{\mathrm{LF}}\right)\right)\right),$$

where BN denotes the batch normalization, MLP denotes the multilayer perceptron, and GAP denotes the global average pooling.

Spatial attention $M_{s}\left(F_{\mathrm{LF}}\right)$ emphasizes or restrains the feature maps in different spatial locations, which mainly includes five steps (Figure 4):

1. 1 × 1 convolutional layer to compress the channel dimensions.
2. Stride convolution and max-pooling layers combined to enlarge the receptive field to receive more useful information.
3. Convolutional group with two 3 × 3 convolutional layers to catch the spatial context information and up-sampling layer to recover the spatial dimensions.
4. 1 × 1 convolutional shortcut and adding its output to the step 3 output to further enrich the spatial context information.
5. 1 × 1 convolutional layer to recover the channel dimensions.

Thus, the spatial attention is computed as

(10) $$M_{s}\left(F_{\mathrm{LF}}\right)=W_{s_{3}}^{1\;\times \;1}\left(Up\left(W_{s_{2}}^{3\;\times \;3}\left(W_{s_{1}}^{3\;\times \;3}\left(Mp\left(W_{s}^{\mathrm{stride}}\left(W_{s_{1}}^{1\;\times \;1}\left(F_{\mathrm{LF}}\right)\right)\right)\right)\right)\right)+W_{s_{2}}^{1\;\times \;1}\left(W_{s_{1}}^{1\;\times \;1}\left(F_{\mathrm{LF}}\right)\right)\right),$$

where Up denotes up-sampling, and Mp denotes max-pooling.

### 3.3. Wavelet-Based Loss Function

In order to balance the reconstructing depth maps by minimizing the difference between the ground truth while also penalizing the loss of high-frequency details that typically correspond to the object boundaries in the scene, four loss terms were combined in our loss function as follows:

1. Depth loss. Balance loss contributions for different distances. We calculate the BerHu loss [31] in logarithm space:
  (11) $$L_{\mathrm{dep}}=\frac{1}{n}\sum_{i=1}^{n}\ln\left({\left|g_{i}-d_{i}\right|}_{b}+\alpha_{1}\right),$$
  where
  (12) $${\left|x\right|}_{b}=\{\begin{array}{c} \left|x\right|,\;\;\left|x\right|\leq c\\ \frac{x^{2}+c^{2}}{2c},\;\;\left|x\right|>c \end{array},$$
  $d_{i}$ and $g_{i}$ are the predicted depth map value and corresponding ground truth for pixel index i, respectively, *n* is the total number of pixels in the current batch, $\alpha_{1}\;$= 5 is a constant parameter; and we set $c=0.2\;\underset{n}{\max}\left(\left|g_{i}-d_{i}\right|\right)$.
2. Gradient loss. Penalizes acute object boundary changes in both the x and y directions that show abundant fine-feature granularity:
  (13) $$L_{\mathrm{gra}}=\frac{1}{n}\sum_{i=1}^{n}\ln\left(\left|\nabla_{x}^{\mathrm{sobel}}\left(e_{i}\right)\right|+\left|\nabla_{y}^{\mathrm{sobel}}\left(e_{i}\right)\right|+\alpha_{2}\right),$$
  where e is the $L_{1}$ Euclidean distance between the predicted depth map and the corresponding ground truth, $\nabla_{x}^{\mathrm{sobel}}$ and $\nabla_{y}^{\mathrm{sobel}}$ represent the horizontal and vertical Sobel operators that calculate the gradient information, and $\alpha_{2}\;$= 0.5 is a constant parameter.
3. Normal loss. Minimize the angle between the predicted surface normal and corresponding ground truth to help emphasize the small details in the predicted depth map:
  (14) $$L_{\mathrm{nor}}=\frac{1}{n}\sum_{i=1}^{n}\left|1-\frac{\langle n_{i}^{d},n_{i}^{g}\rangle }{\sqrt{\langle n_{i}^{d},n_{i}^{d}\rangle }\sqrt{\langle n_{i}^{g},n_{i}^{g}\rangle }}\right|,$$
  where $n_{i}^{d}=\left[-\nabla_{x}\left(d_{i}\right),-\nabla_{y}\left(d_{i}\right),1\right]$ and $n_{i}^{g}=\left[-\nabla_{x}\left(g_{i}\right),-\nabla_{y}\left(g_{i}\right),1\right]$ are the surface normal for the predicted depth map and corresponding ground truth, respectively.
4. SSIM loss. Global consistency metric commonly employed for computer vision tasks:
  (15) $$L_{\mathrm{SSIM}}=1-\frac{\left(2\mu_{d}\mu_{g}+c_{1}\right)\left(2\delta_{dg}+c_{2}\right)}{\left(\mu_{d}^{2}+\mu_{g}^{2}+c_{1}\right)\left(\delta_{d}^{2}+\delta_{g}^{2}+c_{2}\right)},$$
  where $\mu_{d}$ and $\mu_{g}$ are the predicted depth map and ground truth means, respectively, $\delta_{d}$ and $\delta_{g}$ are predicted depth map and ground truth standard deviations, respectively, $\delta_{dg}$ is the covariance between the predicted depth map and ground truth, and constants $c_{1}$ = 2 and $c_{2}$ = 6 follow [46].

**Figure 5:**
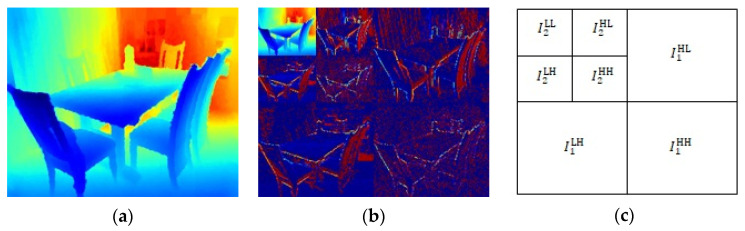
Discrete wavelet transform (DWT) process for depth maps, with two iterations for example: (**a**) original depth map, (**b**) depth map after 2 DWT iterations, and (**c**) labels for different image patches.

Given the DWT invertibility, all depth maps features are preserved by the decomposition scheme. Importantly, DWT captures the depth map location and frequency information, which is helpful for penalizing the high-frequency detail loss that typically corresponds with the object texture. Thus, we propose combining the DWT and multiple loss terms. Figure 5 shows applying iterative DWT decomposes the depth map into different sub-band images, which can be expressed as

(16) $$I_{i+1}^{\mathrm{LL}},I_{i+1}^{\mathrm{LH}},I_{i+1}^{\mathrm{HL}},I_{i+1}^{\mathrm{HH}}=\mathrm{DWT}\;\left(I_{i}^{\mathrm{LL}}\right),$$

where subscript i refers to output from the i-th DWT iteration, and $I_{0}^{\mathrm{LL}}$ is the original depth map.

The four loss terms described above are calculated from the original depth map, $I_{0}^{\mathrm{LL}}$, and sub-band images $I_{i}^{\mathrm{LL}}$,i=1,⋯,n, where n is the number of DWT iterations. We supplemented some depth losses on the basis of the sub-band images $I_{i}^{\mathrm{LH}},I_{i}^{\mathrm{HL}},$ and $I_{i}^{\mathrm{HH}},\;i=1,\cdots ,n$, i.e., loss information for high-frequency details that typically correspond to the object’s horizontal edge, vertical edge, and corner in the depth map, which are very useful for fine-grain estimation. These loss terms can be expressed as

(17) $$L_{W-\mathrm{dep}}=\sum_{i=0}^{n}L_{\mathrm{dep}}\left(\;I_{i}^{\mathrm{LL}}\right)+\sum_{i=1}^{n}\left(L_{\mathrm{dep}}\left(\;I_{i}^{\mathrm{LH}}\right)+L_{\mathrm{dep}}\left(I_{i}^{\mathrm{HL}}\right)+L_{\mathrm{dep}}\left(I_{i}^{\mathrm{HH}}\right)\right),$$

(18) $$L_{W-\mathrm{gra}}=\sum_{i=0}^{n}L_{\mathrm{gra}}\left(\;I_{i}^{\mathrm{LL}}\right),$$

(19) $$L_{W-\mathrm{nor}}=\sum_{i=0}^{n}L_{\mathrm{nor}}\left(\;I_{i}^{\mathrm{LL}}\right),$$

and

(20) $$L_{W-\mathrm{SSIM}}=\sum_{i=0}^{n}L_{\mathrm{SSIM}}\left(\;I_{i}^{\mathrm{LL}}\right),$$

and hence, the final loss function is

(21) $$L_{\mathrm{total}}=L_{W-\mathrm{dep}}+L_{W-\mathrm{gra}}+L_{W-\mathrm{nor}}+L_{W-\mathrm{SSIM}}.$$

Similar conclusions were found by [15] and [46]. Reference [46] extended the SSIM loss by combining it with DWT and showed that this simple modification could improve reconstruction for single-image dehazing. Reference [15] showed that simply allocating larger weights to edge areas in the loss function could boost performances in the border areas.

## 4. Experiments

Section 4.1 describes the experimental setup, including the datasets, evaluation metrics, and implementation details. Section 4.2 compares the experimental results with the current state-of-the-art methods on two public datasets: NYU-Depth-V2 [50] (indoor scenes) and KITTI [51] (outdoor scenes). Section 4.3 uses the NYU-Depth-V2 dataset to analyze the effectiveness and rationality of the AFDB and wavelet-based loss function. Finally, Section 4.4 uses cross-dataset validation on the iBims-1 [52] dataset to assess the proposed method’s generality.

### 4.1. Experimental Setup

#### 4.1.1. Datasets

The NYU-Depth-V2 dataset contains 464 indoor scenes captured by Microsoft Kinect devices. Following the official split, we used 249 scenes (approximately 50-K pair-wise images) for training and 215 scenes (654 pair-wise images) for testing.

The KITTI dataset was captured using a stereo camera and rotating LIDAR sensor mounted on a moving car. Following the commonly used Eigen split [30], we used 22-K images from 28 scenes for training and 697 images from different scenes for testing.

iBims-1 is a high-quality RGBD dataset comprising 100 high-quality images and corresponding depth maps particularly designed to test MDE methods. A digital single-lens reflex camera and high-precision laser scanner were used to acquire the high-resolution images and highly accurate depth maps for diverse indoor scenarios. We use iBims-1 for cross-dataset validation to assess the proposed method’s generality.

#### 4.1.2. Evaluation Metrics

The performance was quantitatively evaluated using standard metrics for these datasets, as shown below for the ground truth depth $y_{i}^{*}$, estimated depth $y_{i}$, and total pixels n in all evaluated depth maps.

- Absolute relative difference (Abs Rel):
  (22) $$\mathrm{Abs}\;\mathrm{Rel}=\frac{1}{n}\sum_{i}\frac{\left|y_{i}-y_{i}^{*}\right|}{y_{i}^{*}}.$$
- Squared relative difference (Sq Rel):
  (23) $$\mathrm{Sq}\;\mathrm{Rel}=\frac{1}{n}\sum_{i}\frac{\left\|y_{i}-y_{i}^{*}{}^{2}\right\|}{y_{i}^{*}}.$$
- Mean Log10 error (log10):
  (24) $$\log10=\frac{1}{n}\sum_{i}\left|\log_{10}y_{i}-\log_{10}y_{i}^{*}\right|.$$
- Root mean squared error (RMS):
  (25) $$\mathrm{RMS}=\sqrt{\frac{1}{n}\sum_{i}{\left(y_{i}-y_{i}^{*}\right)}^{2}}.$$
- Log10 root mean squared error (logRMS):
  (26) $$\mathrm{logRMS}=\sqrt{\frac{1}{n}\sum_{i}{\left(\log_{10}y_{i}-\log_{10}y_{i}^{*}\right)}^{2}}.$$
- Threshold accuracy (TA):
  (27) $$\mathrm{TA}=\frac{1}{n}\sum_{i}g\left(y_{i},y_{i}^{*}\right),$$
  where
  (28) $$g\left(y_{i},y_{i}^{*}\right)=\{\begin{array}{l} 1,\;\;\delta =\max\left(\frac{y_{i}^{*}}{y_{i}},\frac{y_{i}}{y_{i}^{*}}\right)<\mathrm{thr}\\ 0,\;\;\mathrm{otherwise} \end{array}.$$

The threshold accuracy is the ratio of the maximum relative error δ below the threshold thr. Conditions δ< 1.25, δ< 1.25^2^, and δ< 1.25^3^ were used in the experiment, denoted as $\delta_{1}$, $\delta_{2}$, $\mathrm{and}\;\delta_{3}$, respectively.

#### 4.1.3. Implementation Details

The proposed model was implemented with the PyTorch [53] framework and trained using two Nvidia RTX 2080ti graphics processing units (GPUs). The encoders were both pretrained on the ImageNet dataset [54], and the other layers were randomly initialized. The Adam [55] optimizer was selected with β_1_ = 0.9 and β_2_ = 0.999, and the weight decay = 0.0001. We set the batch size = 16 and trained the model for 20 epochs.

For the NYU-Depth-V2 dataset, we first cropped each image to 228 × 304 pixels, and the offline data augmentation methods were as the same as those of the mainstream approaches [18,20,22], i.e., each training image was augmented with random scaling (0.8, 1.2), rotation (−5°, 5°), horizontal flip, rectangular window dropping, and color shift (multiplied by random value (0.8, 1.2)).

For the KITTI dataset, we masked out the sparse depth maps projected by the LIDAR point cloud and evaluated the predicted results only for valid points with ground depths. We capped the maximum estimation at the KITTI dataset maximum depth (80 m). The data augmentation methods were the same as those in [23].

### 4.2. Results

**Figure 6:**
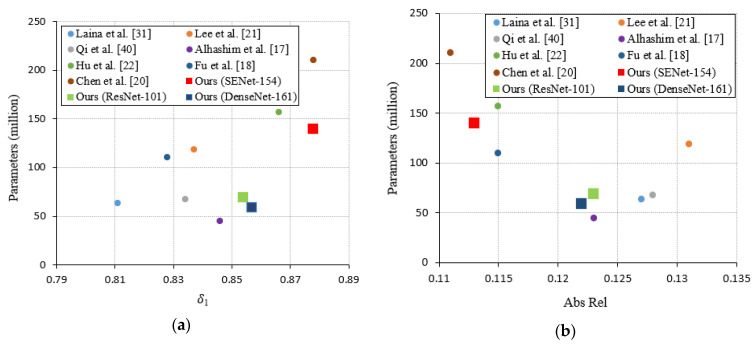
Model parameters and performance (**a**) with respect to $\delta_{1}$ and (**b**) with respect to the absolute relative difference (Abs Rel).

Table 1 shows the evaluation metrics comparing the proposed model with several state-of-the-art methods on NYU-Depth-V2. The DenseNet-161, ResNet-101, and SENet-154 encoders were selected to verify the proposed method’s flexibility. Figure 6 visualizes the trade-off between the performance and model parameters. The results for the comparison methods were taken from their relevant literature.

Table 1 confirms that the proposed method achieved good performances for all the encoder architectures, with the SENet-154 encoder architecture providing the best performance. The proposed method also achieved a comparable or better performance compared with the current state-of-the-art methods.

Figure 6 shows that the proposed model achieved better a trade-off between the performance and model parameters, with only the Abs Rel metric being less than [20], but [20] has more parameters. The proposed method with the DenseNet-161 and ResNet-101 encoders achieved better performances compared with other methods with less than 100 M parameters.

**Figure 7:**
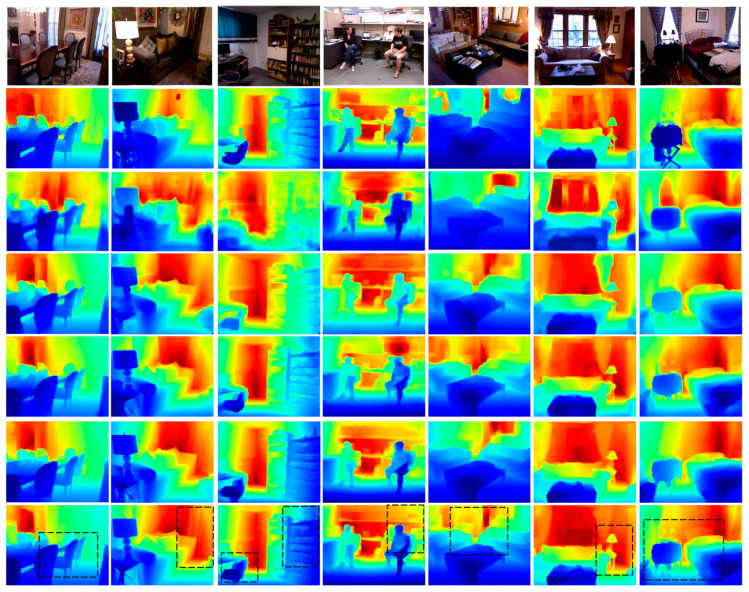
Qualitative evaluations on NYU-Depth-V2. Rows from top to bottom: original RGB images, ground truth depth maps, Laina et al. [31], Alhashim et al. [17], Hu et al. [22], Chen et al. [20], and the proposed method. Regions in black boxes highlight the better-predicted results. Color indicates depth, where red is far and blue is close.

Figure 7 compares the estimated depth maps, and more qualitative results are presented in Appendix A. The display pixels for all the estimated depth maps were the same as those for ground truth to provide easier comparisons. The proposed method achieved better geometric details and object boundaries than the other methods. Thus, the proposed method provides better fine-grain estimations.

**Figure 8:**
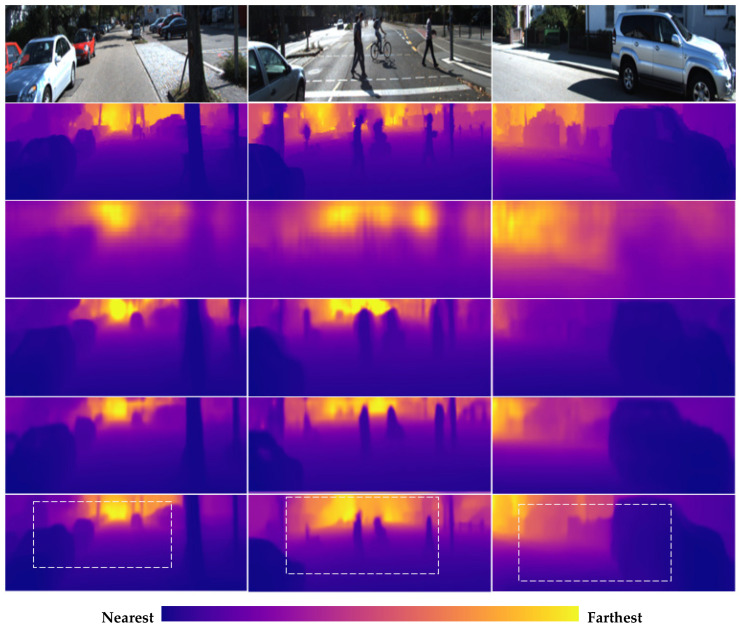
Qualitative evaluations on the KITTI dataset. Rows from top to bottom: original RGB images, ground truth depth maps, Eigen et al. [30], Godard et al. [14], Chen et al. [23], and the proposed method. Regions in the white boxes highlight the better-predicted results. The ground truth maps were interpolated from the sparse measurements for better visualization. Color indicates depth; yellow is far, and purple is close. We set the colors of all outdoor depth maps in our work according to the distance, as in the color bar above.

Table 2 compares the proposed method on the KITTI test dataset using the SENet-154 encoder, with some quantitative comparisons in Figure 8 and more qualitative results in Appendix A. The proposed method outperforms most state-of-the-art methods and provides better object boundaries.

### 4.3. Algorithm Analysis

We conducted several experiments on NYU-Depth-V2 to investigate the effectiveness and rationality for the proposed AFDB and wavelet-based loss functions with the SENet-154 encoder.

#### 4.3.1. AFDB

**Figure 9:**
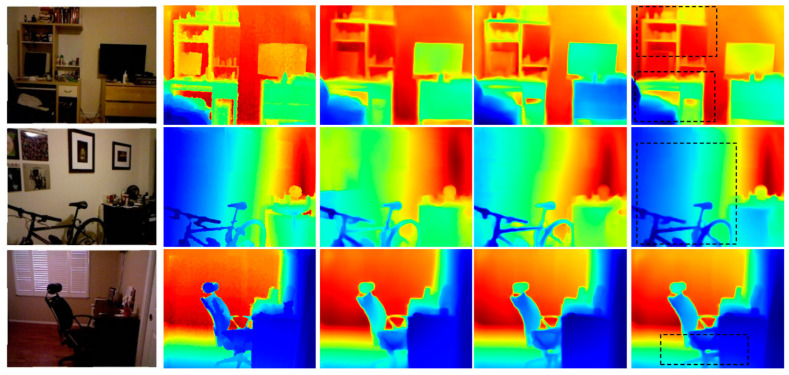
Feature distillation methods on NYU-Depth-V2. Columns from left to right: original RGB images, ground truth depth maps, Hui et al. [43], Liu et al. [44], and proposed approach. Regions in black boxes highlight the better-predicted results. Color indicates depth; red is far, and blue is close.

Figure 9 and Table 3 compare other feature distillation methods with the proposed AFDB. Distillation steps = 4, and DWT iterations = 2 for all evaluations. All metrics are improved for the proposed AFDB at the cost of a few more model parameters. The proposed feature distillation could better predict detailed depth map characteristics.

Table 4 shows the ablation effects, i.e., distillation step and JAM influences, for the prediction results and model performance. We used two DWT iterations to decompose the depth map. More distillation steps can improve the evaluation metrics but increases the model parameters. Almost all evaluation metrics worsened for six or more distillation steps, mainly because five-step distillation generates sufficient features for subsequent treatments, and more steps just increase the local feature fusion burdens. All metrics are improved for the proposed JAM at the cost of a few more model parameters.

#### 4.3.2. Loss Function

Table 5 shows the performance metrics for the proposed model with different loss functions for network training. We gradually added the loss terms described in Section 3.3 to assess the loss terms selection rationality using four-step distillation as the baseline. All evaluation metrics improved with increased loss terms. Thus, the proposed loss function selection method is effective and rational.

Table 6 shows the effects from DWT iterations using the wavelet-based loss function (Equation (21)) to train the network. Three DWT iterations are sufficient to obtain the optimal results. The increased iterations reduce the performance, because the depth map size gradually reduces with the increased iterations, and the detailed depth map features from the smallest scale become indistinct, which may adversely influence the estimation quality.

### 4.4. Cross-Dataset Validation

We performed cross-dataset validation to assess the proposed method’s generality. We used the iBims-1 dataset, because it contains different indoor scenarios and has higher-quality depth maps closer to real depth values compared with NYU-Depth-V2. Therefore, cross-dataset validation on the iBims-1 dataset could verify the model efficiency for different data distributions between training and testing sets. The corresponding evaluation metrics are also more objective and accurate due to the higher precision depth maps.

**Figure 10:**
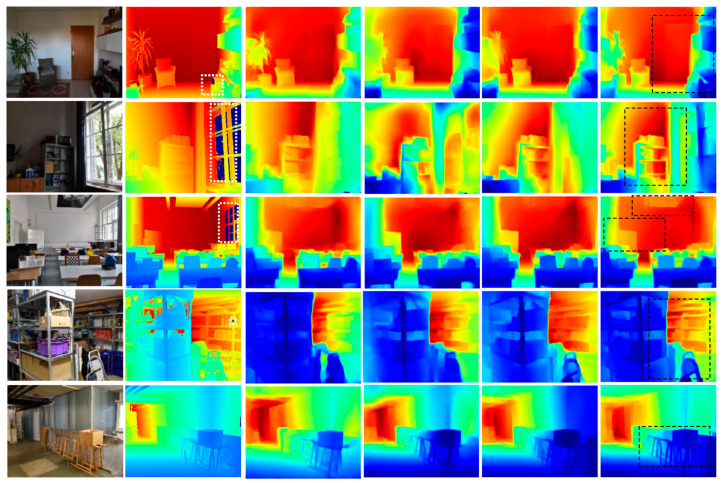
Cross-validation trained on NYU-Depth-V2 and tested on the iBims-1 datasets. Columns from left to right: original RGB images, ground truth depth maps, Alhashim et al. [17], Hu et al. [22], Chen et al. [20], and the proposed method. Regions in white boxes show missing or incorrect depth values from the ground truth data. Regions in black boxes highlight the better-predicted results. Colors indicate depth; red is far, and blue is close.

The proposed network was first trained on NYU-Depth-V2 to generate a pretrained model. Then, the pretrained model was used without fine-tuning to estimate the iBims-1 depth maps. Table 7 shows the corresponding evaluation metrics for iBims-1, and Figure 10 shows some qualitative comparisons. The settings for the compared methods were the same as for the proposed method. The pretrained models for the compared methods were generated by running their open-source codes.

The test results of the pretrained models on iBims-1 were quite different from those on NYU-Depth-V2. In contrast to the earlier comparisons in Table 1, [17] has better performances than [20] and [22]. The proposed model achieved significantly better performances than the three comparative methods. Thus, the proposed method could better estimate the geometric details and object boundaries for these different scenes than the three current state-of-the-art methods.

## 5. Conclusions

This paper proposed a new DCNN for monocular depth estimation. Two improvements were realized compared with previous methods. We made a combination of joint attention and feature distillation mechanisms in the decoder to boost the feature discriminative modulation and proposed a wavelet-based loss function to emphasize the detailed depth map features. The experimental results on the two public datasets verified the proposed method’s effectiveness. The experiments were also conducted to verify the proposed approach effectiveness and rationality. The generality for the proposed model was demonstrated using cross-dataset validation.

Future works will focus on applying the proposed MDE methods to 3D vision applications, such as augmented reality, simultaneous localization and mapping (SLAM), and indoor scene reconstruction.

## Figures and Tables

**Table 1:** Model performance on NYU-Depth-V2. Best scores are highlighted in bold font. The attention-based feature distillation block (AFDB) distillation step = 5 and discrete wavelet transform (DWT) iteration = 3. Abs Rel: absolute relative difference and RMS: root mean squared error.

Method	Error (Lower is Better)			Accuracy (Higher is Better)		
	Abs Rel	RMS	Log10	$\delta_{1}$	$\delta_{2}$	$\delta_{3}$
Eigen et al. [30]	0.212	0.873	-	0.611	0.887	0.969
Laina et al. [31]	0.127	0.573	0.055	0.811	0.953	0.988
Chen et al. [23]	0.138	0.496	-	0.826	0.964	0.990
Lee et al. [21]	0.131	0.538	-	0.837	0.971	0.994
Qi et al. [40]	0.128	0.569	-	0.834	0.960	0.990
Zhao et al. [19]	0.128	0.523	0.059	0.813	0.964	0.992
Li et al. [33]	0.134	0.540	0.056	0.832	0.965	0.989
Hao et al. [26]	0.127	0.555	-	0.841	0.966	0.991
Alhashim et al. [17]	0.123	0.465	0.053	0.846	0.974	0.994
Huang et al. [39]	0.122	0.459	0.051	0.859	0.972	0.993
Hu et al. [22]	0.115	0.530	0.050	0.866	0.975	0.993
Fu et al. [18]	0.115	0.509	0.051	0.828	0.965	0.992
Wang et al. [25]	0.115	0.519	0.049	0.871	0.975	0.993
Chen et al. [20]	**0.111**	0.514	**0.048**	**0.878**	0.977	0.994
Ours (DenseNet-161)	0.122	0.534	0.050	0.857	0.972	0.993
Ours (ResNet-101)	0.123	0.532	0.052	0.854	0.972	0.992
Ours (SENet-154)	0.113	**0.504**	**0.048**	**0.878**	**0.978**	**0.995**

**Table 2:** Performance evaluation on the KITTI. The best scores are highlighted in bold font. Sq Rel: squared relative difference.

Method	Error (Lower is Better)				Accuracy (Higher is Better)		
	Abs Rel	RMS	Sq Rel	logRMS	$\delta_{1}$	$\delta_{2}$	$\delta_{3}$
Eigen et al. [30]	0.190	7.156	1.515	0.270	0.692	0.899	0.967
Godard et al. [14]	0.148	5.927	1.515	0.247	0.802	0.922	0.964
Jiang et al. [27]	0.128	5.299	1.037	0.224	0.837	0.939	0.971
Li et al. [33]	0.104	4.513	0.697	0.164	0.868	0.967	0.990
Liu et al. [13]	0.106	4.274	0.686	0.176	0.878	0.968	0.986
Wang et al. [25]	0.096	4.327	0.655	0.171	0.893	0.963	0.983
Alhashim et al. [17]	0.093	4.170	0.589	0.171	0.886	0.965	0.986
Chen et al. [23]	0.083	3.599	0.437	0.127	0.919	0.982	**0.995**
Fu et al. [18]	0.072	**2.727**	0.307	**0.120**	0.932	**0.984**	0.994
Ours (SENet-154)	**0.071**	2.848	**0.306**	0.121	**0.933**	0.983	**0.995**

**Table 3:** Feature distillation performance on NYU-Depth-V2.

Method	Parameters	Error (Lower is Better)			Accuracy (Higher is Better)		
		Abs Rel	RMS	Log10	$\delta_{1}$	$\delta_{2}$	$\delta_{3}$
Hui et al. [43]	127.6 M	0.121	0.515	0.050	0.863	0.973	0.992
Liu et al. [44]	133.1 M	0.114	0.517	0.049	0.871	0.976	0.993
AFDB	135.7 M	0.113	0.509	0.049	0.877	0.978	0.994

**Table 4:** The AFDB performance under different settings. Method subscripts show the distillation steps (w/o means without). JAM: joint attention module.

Method	Parameters	Error (Lower is Better)			Accuracy (Higher is Better)		
		Abs Rel	RMS	Log10	$\delta_{1}$	$\delta_{2}$	$\delta_{3}$
AFDB _3, JAM_	134.4 M	0.117	0.511	0.050	0.870	0.974	0.994
AFDB _4, JAM_	135.7 M	0.113	0.509	0.049	0.877	0.978	0.994
AFDB _5, JAM_	139.2 M	0.113	0.504	0.048	0.878	0.978	0.995
AFDB _6, JAM_	142.7 M	0.121	0.503	0.050	0.867	0.976	0.994
AFDB _4, w/o JAM_	133.9 M	0.117	0.511	0.050	0.867	0.974	0.992

**Table 5:** Proposed method performance for different loss functions. SSIM: structural similarity. Each loss function is defined in Section 3.3.

Loss Function	Error (Lower is Better)			Accuracy (Higher is Better)		
	Abs Rel	RMS	Log10	$\delta_{1}$	$\delta_{2}$	$\delta_{3}$
$L_{\mathrm{dep}}$	0.121	0.534	0.051	0.857	0.970	0.992
$L_{\mathrm{dep}}$+$L_{\mathrm{gra}}$	0.117	0.525	0.050	0.865	0.975	0.993
$L_{\mathrm{dep}}$+$L_{\mathrm{gra}}$+$L_{\mathrm{nor}}$	0.116	0.521	0.050	0.868	0.976	0.993
$L_{\mathrm{dep}}$+$L_{\mathrm{gra}}$+$L_{\mathrm{nor}}$+$L_{\mathrm{SSIM}}$	0.114	0.515	0.049	0.872	0.976	0.994

**Table 6:** DWT iteration effects on the model performance using the wavelet-based loss function.

DWT Iterations	Error (Lower is Better)			Accuracy (Higher is Better)		
	Abs Rel	RMS	Log10	$\delta_{1}$	$\delta_{2}$	$\delta_{3}$
One	0.114	0.509	0.049	0.874	0.975	0.994
Two	0.113	0.509	0.049	0.877	0.978	0.994
Three	0.113	0.504	0.048	0.877	0.978	0.994
Four	0.114	0.509	0.049	0.873	0.976	0.994

**Table 7:** Cross-dataset validation trained on NYU-Depth-V2 and tested on the iBims-1 dataset.

Method	Error (Lower is Better)			Accuracy (Higher is Better)		
	Abs Rel	RMS	Log10	$\delta_{1}$	$\delta_{2}$	$\delta_{3}$
Alhashim et al. [17]	0.346	2.772	0.199	0.179	0.547	0.827
Hu et al. [22]	0.360	2.815	0.208	0.162	0.497	0.816
Chen et al. [20]	0.349	2.750	0.200	0.162	0.531	0.849
Ours	0.329	2.665	0.184	0.192	0.601	0.876

**Figure A1:**
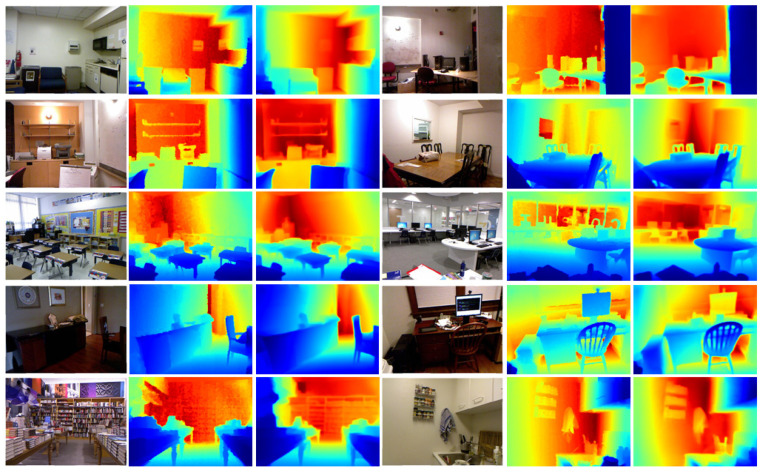
Example outcomes for the proposed method on NYU-Depth-V2. Columns from left to right: original RGB images, ground truth depth maps, and proposed model predicted depth maps. Colors indicate depth; red is far, and blue is close.

**Figure A2:**
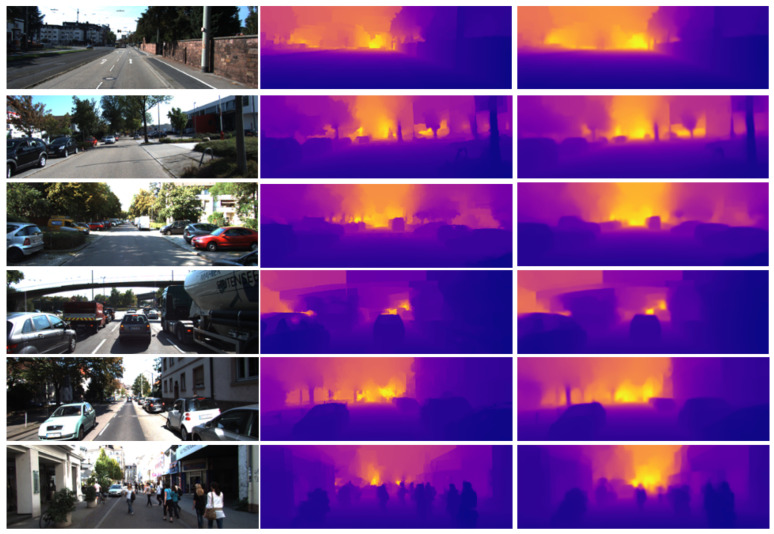
Example outcomes for the proposed method on KITTI. Columns from left to right: original RGB images, ground truth depth maps, and proposed model predicted depth maps. Colors indicate depth; yellow is far, and purple is close. Ground truth maps were interpolated from sparse measurements for better visualization.

## Data Availability

The data presented in this study are available on request from the corresponding author.
